# Training the next generation of Africa’s doctors: why medical schools should embrace the team-based learning pedagogy

**DOI:** 10.1186/s12909-019-1845-y

**Published:** 2019-11-04

**Authors:** Charles Okot Odongo, Kristina Talbert-Slagle

**Affiliations:** 1 Department of Pharmacology & Therapeutics, Faculty of Medicine, Gulu University, P.O. Box 166, Gulu, Uganda; 2 Department of Internal Medicine, Yale School of Medicine, P.O. Box 208364, New Haven, CT 06510 USA

**Keywords:** Team-based learning, Africa, Medical schools, Doctors, Pedagogy

## Abstract

**Background:**

As far back as 1995, the Cape Town Declaration on training Africa’s future doctor recognized the need for medical schools to adopt active-learning strategies in order to nurture holistic development of the doctor. However, medical education in Africa remains largely stuck with traditional pedagogies that emphasize the ‘hard skills’ such as knowledge and clinical acumen while doing little to develop ‘soft skills’ such as effective communication, teamwork, critical thinking or life-long learning skills.

**Body of abstract:**

By reviewing literature on Africa’s epidemiologic and demographic transitions, we establish the need for increasing the output of well-trained doctors in order to match the continent’s complex current and future healthcare needs. Challenges that bedevil African medical education such as outdated curricula, limited educational infrastructure and chronic resource constraints are presented and discussed. Furthermore, increased student enrollments, a trend observed at many schools, coupled with chronic faculty shortages have inadvertently presented specific barriers against the success of small-group active-learning strategies such as Problem-Based and Case-Based Learning. We argue that Team-Based Learning (TBL) offers a robust alternative for delivering holistic medical education in the current setting. TBL is instructor-driven and embodies key attributes that foster development of both ‘hard’ and ‘soft’ skills. We elaborate on advantages that TBL is likely to bring to the African medical education landscape, including increased learner enthusiasm and creativity, accountability, peer mentorship, deep learning and better knowledge retention. As with all new pedagogical methods, challenges anticipated during initial implementation of TBL are discussed followed by the limited pilot experiences with TBL in Africa.

**Conclusion:**

For its ability to enable a student-centered, active learning experience delivered at minimum cost, we encourage individual instructors and African medical schools at large, to adopt TBL as a complementary strategy towards realizing the goal of training Africa’s fit-for-purpose doctor.

## Background

Over the years, medical schools globally have experimented with new curricula and different pedagogical formats aimed at improving the learning process [1, 2]. In many settings, implementation of these innovations has not been without challenges, often resulting in substantial modifications or abandonment [2–4]. In some instances, these modifications have appeared to erode key philosophical tenets on which such innovations are premised, and possibly undermining learning outcomes [5, 6]. From its inception in the 1960s, problem-based learning (PBL), arguably the most widely experimented curriculum innovation, has generated plenty of controversy and debate [5–12]. In Africa and probably elsewhere, experiments with PBL in medical education have exposed systemic weaknesses that call into question the sustainability and success of similar innovations going forward [7, 13]. Moreover, resources at many African medical schools are currently outstripped by rising student enrolments [14], a trend likely to compromise educational standards [15]. Thus, medical education in Africa is at a crossroad: in need of reforms that not only improve educational standards but also remain responsive to the continent’s unique challenges and healthcare aspirations [16]. Mindful of lessons from previous experiences with novel pedagogies in Africa [7, 13], this article attempts to vouch for Team-Based Learning (TBL), a form of cooperative learning that is relatively new to medical education [17]. To build our case, information from a wide range of publications on current pedagogical strategies as well as medical education challenges, particularly in Africa were reviewed.

Using Africa’s disease and demographic transition patterns, we justify its physician competency requirements and contrast these with current training experiences at its medical schools. The TBL pedagogical approach is then described showing how it instills core knowledge and skills attributes required to equip Africa’s future doctors with the right competencies for effective practice. We proceed to elaborate how–with considerable parsimony—TBL mitigates the major challenges bedeviling reforms in African medical education. This is followed by examination of bottlenecks anticipated during initial implementation of TBL, together with a review of the limited African pilot experiences.

## Main text

### Disease burden and demographic transitions in Africa

Africa is disproportionately burdened by poverty and disease, a situation compounded by its low level of human resources for health. With an estimated 29% of the global disease burden in 2017 [18], Africa has 3.5% of the global healthcare workforce of which only 1.7% are doctors [19]. At an average of 4.7 children per woman and a population growth rate of 2.7%, Africa’s statistics are the highest globally [20], projecting enormous healthcare needs. Since 1990, life expectancy in Africa has risen steadily - from an average of 49.7 to 60.7 years in 2017 [21]. Despite this improvement, infectious diseases continue to burden millions, coupled with a rising burden of non-communicable diseases such as cancers, diabetes, and cardiovascular diseases [18]. Therefore, Africa’s dual disease epidemic will increasingly require healthcare providers with additional competencies. In recognition of this crisis, health planners have focused on increasing human resources for health [22, 23]. Here, training doctors has been particularly prioritized, not because they will meet the vast projected healthcare needs of the continent, but in the belief that no health system can function optimally without an adequate number of well-trained doctors to provide leadership, mentorship and effective clinical care [22].

### Skills requirement for the next generation of Africa’s doctors

Gone are the days when a doctor sat in the office and dispensed prescriptions with the support of one or two nurses. Doctors are increasingly practicing in teams along with a range of allied and interprofessional scientists. In large referral settings, doctors with varied expertise work collaboratively to deliver high-end interventional care. As heads of clinical teams, doctors are teachers, supervisors, mentors and role models to their subordinates. All these responsibilities require healthy and strong interpersonal relationships. During clinical care, doctor-patient communication needs to be compassionate yet truthful and unambiguous. Very intriguing perhaps, is a new breed of patients who increasingly indulge the web prior to seeking consultation [16], leading to uncomfortable doctor-patient encounters that call for a high level of emotional intelligence. Outside clinical care, doctors serve in managerial positions at all levels of healthcare. As prominent members and opinion leaders, society looks up to them for guidance. Clearly, beyond the ‘hard’ skills, effective delivery on the above mandates require an additional set of skills such as effective communication, compassion, critical thinking and collaboration. Furthermore, doctors require independent learning skills so as to secure their positions as life-long learners capable of keeping pace with new information relevant to their roles. However, until recently, curricula and pedagogical strategies in many African medical schools had barely changed, remaining focused on the transfer of ‘hard’ skills with little regard for ‘soft’ skills [16, 24]. More challenging perhaps, is the fact that soft skills cannot be transferred through instructor-centered, passive learning strategies; rather, they are nurtured through carefully designed pedagogies that afford learners the opportunity to hone these skills through mentored peer interaction. For instance, it is now known that didactic lectures, however eloquent, are less effective for developing problem-solving, critical thinking or independent learning skills [25]. For this reason, regulatory authorities globally are increasingly encouraging the adoption of student-centered active learning strategies in medical education [25–29]. As far back as 1995, the Cape Town Declaration on training Africa’s future doctor recognized the need for medical schools to adopt active-learning strategies [30]. However, reports suggest that sustainable implementation of this recommendation has proved elusive [14, 16, 24, 31].

### Medical education challenges in Africa

The academic environment at most African schools is such that what is termed ‘success’ is typically achieved through individual effort, in competition rather than collaboration with other learners. As most faculty lack formal pedagogical training [16, 24], teaching is mainly done using traditional instructor-centered strategies which more often than not promote learner dependency [14]. Until recently, curricula were generally outdated and in need of reform, yet a significant section of faculty continuously struggle with change initiatives [7, 24]. Despite the availability of rich online resources, the textbook and lecture notes still predominate, in part due to challenges with internet access [31]. Class time is disproportionately didactic, a model premised on the assumption that attendance translates into listening, understanding and knowledge retention. Preclinical students are expected to memorize large amounts of information, which they begin to apply later during clerkship. Academic progress is based primarily on students’ ability to memorize facts, with little credit devoted to other skills. This paradigm continues despite the well-known folly with memory-driven learning - that infrequently used knowledge is quickly forgotten [32, 33]. Moreover, the rate at which medical knowledge is growing is now faster than anyone can comprehend, let alone learn at school. Amidst all this, students lack guidance on effective learning skills, despite increased scientific understanding on the neurobiology of learning [34, 35]. It is no wonder that many students feel overwhelmed by the sheer volume of content to master, a phenomenon that contributes to academic stress [36, 37]. Whereas medical education generally requires considerable personal effort and sacrifice, addressing the above structural challenges in African medical education would significantly enhance learning.

In Africa, medical education is predominantly a preserve of national governments whose competing priorities leave training institutions perennially underfunded [31]. Faculty recruitment and retention remain challenging owing to low public-sector salaries [14]. This situation is compounded by the rising trend in student enrollments that inevitably increases workload for the few faculty [31]. Students, on their part, face numerous challenges, some of which include inadequate learning infrastructure. For instance, Chen et al., highlight space shortages that frustrate students’ efforts to meet for group discussions outside class [14]. Thus, without creative reforms, current challenges are counterproductive to the continent’s aspiration to increase its number of doctors. On a positive note, the teaching and learning environment in some schools has registered modest improvement through the MEPI initiative [38]. More enduring, perhaps, will be the establishment of full-time medical education departments within schools to champion future reforms [39]. Acknowledging that resource constraints will probably persist for some years to come, medical educators need to embrace innovative pedagogies capable of delivering Africa’s ideal doctor [30]. For its ability to optimize learning and enable efficient resource utilization, we posit that TBL is one such strategy that is increasingly gaining favor among medical educators globally [17, 40].

### What is team-based Learning?

TBL is a form of cooperative learning where students are strategically organized into permanent study groups within a single classroom setting where the instructor creates, initiates, and directs all learning activities. The class is operated on 7 core educational principles designed to maximize student preparation and participation while fostering high levels of performance and accountability. Table 1 shows how these principles positively affect learner engagement with course content and peers. Students master content through a three-phase process as follows: Phase 1: pre-class preparation, in which learners consume assigned materials (readings, videos, dissection etc.). Phase 2: readiness assurance to apply new knowledge, achieved when all learners initially take a readiness test individually, followed by retake of the exact same test as a group, and coming to consensus on group answers to each question. This is followed by an instructor-led class debriefing, where groups have the opportunity to make appeals as long as they can advance valid arguments to support their answers. The instructor may also give a short and usually focused lecture, aimed at clarifying misconceptions that become apparent during the debriefing process. In Phase 3, learners have the opportunity to apply new knowledge through group problem-solving using assignments that simulate real-life and often involve complex or ambiguous scenarios. This stage is open-book (or open internet) since it is intended to stimulate guided knowledge acquisition and deeper learning. Groups are given time to confer, after which they make specific choice answers, to be simultaneously reported across the class. TBL assignments should consist of knowledge-application scenarios with potential to generate controversy. Only then can vibrant discussions and deeper learner engagement with content occur [42]. This is particularly true when groups eventually morph into self-managed and highly motivated learning teams capable of using their collective brain power to propose practical solutions to real-life problems [43]. TBL can be applied to teaching single topics or entire courses in higher education [40] and is suitable for both undergraduate and graduate level education [17, 40].

**Table 1:** Core design elements of Team-Based Learning showing their effects on learner engagement with content and peers

Core design element	Rationale/TBL principle	Effect on learner engagement with content	Effect on learner engagement with peers
1. Judicious team formation performed by instructor	Optimal team size and intellectual resources (brain power) to be distributed equally across teams. This does not typically occur when learners are allowed to form their own teams.	Teams with too few learners (e.g. less than 5) lack sufficient ‘intellectual assets’ to tackle complex problems; too many learners (e.g. more than 8) permits ‘social loafing’	Team motivation to work together increases when learners believe their collective brain power matches that of other teams
2. Readiness Assurance	Allows the instructor and team members to verify that all learners are prepared to apply course concepts to solve real-world, or complex tasks.	Individual and team accountability motivates learners to prepare by acquiring background knowledge before coming to class	During group discussions, learners teach each other, often using language that is more familiar than that of the instructor
3. Immediate feedback	Immediate feedback enhances both individual learning and team communication processes by allowing teams to constantly assess the effectiveness of their problem-solving and communication strategies.	Obtaining answers to questions following the group test allows individual misconceptions to be clarified before they are entrenched	Reinforces to team members the value of collaboration. Also provides a disincentive for poor team communication behavior (e.g. poor listening or overassertiveness)
4. Sequencing of in-class problem solving	Proper sequencing of activities- i.e. intrateam followed by interteam activities; enables learners to deepen their level of thinking and can positively affect the team development process	Multiple opportunities to discuss and apply knowledge to solve a problem fosters greater depth of engagement with course concepts and promotes long-term knowledge retention ^46^	Interteam discussions solidify group identity and cohesiveness. Teams want to use their intrateam discussion time effectively to avoid embarrassment during interteam discussions
5. The four Ss	Attention to the 4S structure (i.e. significant problem, same problem, specific choice, simultaneous reporting) fosters individual and team motivation, a common frame of reference, critical thinking and conceptual depth, and vigor during whole class discussions	A significant problem with real-life relevance increases interest during team discussions. Same problem for all teams increases interest during interteam discussions. The requirement to make a specific choice fosters conceptual depth in intra- and interteam discussions.	Simultaneous reporting of specific choice enhances recognition of controversy across teams. Constructive controversy across teams motivates collaboration within teams to defend points of view ^41^
6. Incentive structure	As in any teaching endeavor, the incentive structure has powerful effects on the achievement of course goals	Grading individual performance motivates out-of-class preparation	Grading team performance provides a clear incentive to maximize collaboration
7. Peer evaluation	This is especially critical in a longitudinal TBL curriculum. Feedback from peers may have effects that other forms of feedback may not because peers have a unique relationship with each other as learners	The possibility of a negative review from peers motivates learners to prepare for and participate in class. Peer feedback also shapes specific learner behavior such as over assertiveness and collaboration	Promotes individual learners’ accountability to the team. It also reinforces the importance of every individual’s preparation and participation, as these affect overall team performance.

(Adapted with permission from Haidet et al. [41])

### Team-based Learning brings unique advantages to Africa’s medical education landscape

TBL is an instructor-led pedagogy that stimulates deductive learning within interactive small groups. In contrast to didactic learning strategies, where knowledge-acquisition is largely passive, TBL’s in-class assignments ensure that very early on, learners develop a clear sense of how new knowledge may be applied in real-life. The instructor’s role shifts from knowledge presenter to that of content preparation and moderator of the learning process. Learners are challenged to apply new knowledge in an environment that is competitive yet collaborative, where real-time feedback and guidance are assured. Such environments promote learner enthusiasm and creativity, critical thinking and deeper learning [3, 44], a hallmark of TBL. Furthermore, through teamwork and timely feedback from the instructor, TBL guards against entrenchment of misconceptions that all too often characterize didactic learning strategies. Additionally, repeated testing, using assignments centered around newly-learned concepts provides a powerful boost to knowledge retention. Here, TBL leverages on the most current evidence on learning theory – that knowledge retention is better with instructional strategies that involve repeated testing and not repeated study [35, 45, 46].

With TBL, one can steer multiple teams concurrently without losing the benefit of instructor-guided small group learning models. In North America, experienced instructors have managed classes of up to 200 students [47]. The ability to engage large classrooms in learning and consolidating new concepts should make TBL appealing to African medical schools that aspire to increase enrollment but are financially constrained, making it hard to correspondingly increase faculty [24, 31]. Trials with the problem-based learning curriculum in Zimbabwe failed, in part, due to the high student-to-faculty ratio [13]. Similar challenges have been reported elsewhere [7, 48]. With TBL, schools can increase enrollment and yet deliver a student-centered active learning experience even with few faculty. Moreover, few but dedicated faculty stand a higher chance at negotiating better remuneration which would further boost faculty morale, productivity and learner satisfaction.

Unlike other group-based learning formats (e.g. Case-Based Learning and PBL), where educational resources (e.g. space, instructors, multimedia equipment etc.) are replicated for each group, TBL enables resource economization. For instance, multiple teams working within a single classroom can share space and equipment making substantial savings on scarce institutional resources. Furthermore, when instructors assume primary responsibility for sourcing study materials (notes, videos, research articles etc.), learners can focus on studying assigned material without losing quality time sifting through libraries or scouring the web. In Africa, such activities all too often occur within a backdrop of weak information systems infrastructure that only serves to frustrate learner experiences [14]. Thus, TBL ensures fair access to core study materials while reserving learners’ prerogative to obtain additional information at convenience. Because students bear initial responsibility to read and comprehend new knowledge (pre-class preparation), TBL nurtures independent and life-long learning skills. This is more so because peers as well as the instructor, provide the cognitive support required for learner self-evaluation and improvement in study skills over time.

TBL provides a robust accountability model involving all parties to the learning process. Through the readiness assurance process, learners are accountable for attending class as well as preparing before they come. Peer evaluation (see Table 1) ensures that learners invest time and effort working within their teams. The instructor is accountable for initiating and guiding the learning process, providing cognitive support as learners work through the nuances akin to real-life decision-making. Team discussions involve peer tutoring, which leverage on learners’ ability to use language (or examples) that peers easily relate with. It has been noted that even introverts develop the confidence to speak up, boosting their communication skills [49]. Moreover, intrateam dialogue taps into the diverse perspectives of learners – all of which inform and enrich problem-solving and foster critical thinking. With diverse perspectives, team members are bound to disagree, resulting in passionate rebuttals in defense of one’s point of view. Thus, in addition to honing problem-solving and critical thinking skills, students develop the social skills necessary to harmoniously manage intellectual conflict. Furthermore, by working on the same problem and requiring teams to make specific choices (to be reported simultaneously), team cohesiveness and collaboration are fostered. The benefits of collaboration become evident when team scores turn up higher than the best individual scores on most occasions [50]. Bleakley and Brennan showed that curricula that use team learning strategies produce graduates who are not only competent in the “hard skills” but tend to outperform their peers in areas such as collaboration, tolerance of ambiguity and confidence in practice [51]. In addition, they showed that teamwork reduced the risk of medication errors resulting in better patient care [51]. These observations suggest that TBL learners gain insights into their own strengths and weaknesses, enabling them to negotiate and develop respectful and constructive working relationships.

### Challenges with initial implementation of TBL in a new setting

As with all teaching methods applied to new physical or cultural environments, initial implementation of TBL comes with challenges. These challenges are, however, not unique to resource-constrained settings but may be more pronounced in settings that are not accustomed to change. For instance, context-specific factors such as differences in curriculum orientation or level of instructor autonomy within a course may limit instructors’ freedom to experiment with TBL. In some cases, effective use of TBL will require redesigning entire courses. This can be quite challenging but achievable with gradual effort and determination [52]. Some content may require re-organization into major subunits in order to permit holistic discussion and real-life knowledge application on the subject matter. In pharmacology for instance, course topics under *antibacterial chemotherapy* may have to be merged and covered together without distinct sessions focused on different antibiotic categories. This creates the best platform to tackle assignments that closely model real-life situations e.g. why one antibiotic would be favored over another in managing an infant with bacterial pneumonia. Learners can meaningfully ponder on the different options using diverse arguments drawing on different aspects of the case such as pathophysiology, patient factors (e.g. age, immune status) or social-economic considerations and how they interplay with basic pharmacodynamic or pharmacokinetic principles. Likewise, in *HIV pharmacotherapy*, study materials covering its virology together with the basic and clinical science behind anti-retroviral therapies should be studied jointly in order to prepare learners for knowledge application assignments modeled on real-life challenges.

Developing appropriate knowledge application assignments is another challenge, since assignments should be relevant and captivating with ability to elicit the right learning experiences. Well-crafted assignments should model the challenges, nuances and ambiguities commonly encountered in practice, and not simply require solutions discoverable by re-examining prior assigned readings. Effective assignments should spur intellectual conflict, fostering deeper engagement with new knowledge through dialogue, interpretation, or extrapolation. Such assignments provoke probing questions and rebuttals during discussions, since each team is required to make (and later defend) a specific position lest they are embarrassed before the class. Only then can assignments evoke the vibrant discussions capable of yielding productive and enjoyable outcomes for all. Haidet and colleagues have used the term “constructive controversy” to sum up the characteristics of such assignments [41]. In contrast, simple and straightforward assignments may, at most, elicit intragroup dialogue, but risk stifling intergroup discussion as different teams will probably reach the same specific conclusion. Such assignments make TBL appear like any ordinary quiz session. Experts propose the use of the ‘backward design’ approach while developing appropriate assignments [53]. It is important that sufficient time (e.g. 50% of class-time) is devoted to knowledge-application (phase 3) as this ensures multiple opportunities for ‘learning moments’ that reinforce key concepts and contribute towards achieving course objectives [52].

The fact that each class begins with a test makes TBL stressful to some learners who may detest the anxiety associated with pre-class preparation. However, this is probably the most important phase in TBL as no amount of team discussion can compensate for lack of preparation, moreover, such ill-prepared members risk slowing team progress during problem-solving. Despite such stress, learning moments that result from discovering your own shortcomings during team discussions (or feedback) can be truly rewarding, making the pre-class effort worthwhile. Discovering one’s own shortcomings helps learners hone skills such as critical appraisal, and the knowledge gained subsequently is better retained as studies show [46].

TBL ensures that individual and group effort are appropriately rewarded through judicious course assessment. Unlike other small group learning formats, low performers do not necessarily pull down the scores of high performers. This is achieved using a grading scheme in which each learners’ final score (per session), is computed as an average between individual and group scores in the readiness assurance test. Each learner is thus held accountable for their own final score. Also, the closer learners work together, the better their team score, thus improving everyone’s final score [54]. It is important to ensure that TBL scores count significantly towards final course grades as this provides additional impetus for adequate pre-class preparation, and subsequently, a vibrant class experience. Compared with lecture-based methods, there is evidence that academic performance with TBL is similar, if not better [50, 55]. Nevertheless, the thrust of our case for TBL lies in its ability to nurture soft skills through cooperative learning implemented in a manner that is cost-effective for resource-poor settings. Due to increased popularity in recent times, TBL hybrids, where one or more of the aforementioned steps are omitted or modified, have been implemented and reported. While this may be justifiable for practical or context-specific reasons, it is important to note that such modifications can have significant effects on class outcomes as some of the underlying principles laid out in Table 1 may be eroded. For this reason, Haidet *et.al.* have argued for standardization of scholarly reports on TBL experiences [41]. Where such modifications have occurred, such standardized reporting would allow for more meaningful interpretation, comparisons, critiquing or replication of studies.

### Some early African experiences with team-based learning in medical education

There have been few trials with TBL in Africa. One pilot was done in Zimbabwe between 2010 and 12, during a time when the declining economic situation imposed severe financial strain at the University of Zimbabwe, leading to a 69% vacancy rate at the College of Health Sciences [56]. Investigators implemented TBL to deliver two cycles of an HIV/AIDS module to 5th year medical students within a competency-based curriculum. At the end of each cycle, students completed a survey that assessed their experiences and perceived knowledge gain. All respondents agreed that TBL was an effective way to learn about HIV, with 66% strongly agreeing. The majority (94%) felt TBL was more stimulating than lectures, while 91 and 94% respectively, felt it fostered enthusiasm for course material and collaboration. Students perceived significant improvements in knowledge gained, particularly with challenging scenarios such as managing HIV drug resistance and treatment of HIV-TB co-infection. The authors concluded that TBL was feasible and offered a promising and exciting way of providing high-quality medical education at resource-limited institutions. In Tanzania, TBL was piloted with an ectoparasite module at Kilimanjaro Christian Medical University College in 2012 [57]. Here, investigators sought to explore perceptions as well as improvement in examination scores in comparison to the previous year, where a didactic approach was employed for the same course. For the 158 students surveyed, the mean score across six domains (content, learning process, knowledge, educational environment, assessment, instructors & efficiency) ranged from 4.2–4.5 (5 being the most favorable). Using a strength of consensus measure, there was a high degree of consensus among learners (85–90%). An improvement in examination scores was also noted, particularly among the lower 50%. Elsewhere, such improved performance among the “at risk” group has been attributed to the strong social and academic support (through peer tutoring) embodied within TBL [55, 58, 59]. Overall, these studies attest to the applicability of TBL within the African context. We recently conducted a similar pilot at the University of Liberia’s A.M. Dogliotti College of Medicine, which faces similar infrastructural and human resource challenges. Our initial experience with TBL will be reported elsewhere.

## Conclusion

Since its initial use in the health sciences in 1999 [60], global interest in TBL has increased due to its ease of adoption as well as its consistency with current accreditation standards that seek to promote active learning strategies [25–28]. It is no wonder that world-wide, medical schools are increasingly adopting TBL as an instructional method in some shape or form for both preclinical and clinical education [40, 50]. As Africa strives to maintain its credibility in this highly dynamic field [16], TBL presents a resource-efficient avenue for imparting both hard and soft skills critical for effective clinical practice in the twenty-first century. We therefore urge individual instructors as well as African medical schools at large, to experiment with TBL as a way to strengthen medical education in a manner that is responsive to human and institutional resource constraints.

## Data Availability

Data sharing is not applicable to this article as no datasets were generated or analyzed during the current study.

## Abbreviations

MEPI: Medical Education Partnership Initiative
PBL: Problem-Based Learning
TBL: Team-Based Learning
